# Disaster preparedness and cultural factors: a comparative study in Romania and Malta

**DOI:** 10.1111/disa.12433

**Published:** 2020-11-06

**Authors:** Sandra Appleby‐Arnold, Noellie Brockdorff, Ivana Jakovljev, Sunčica Zdravković

**Affiliations:** ^1^ Knowledge Exchange and Impact Manager at the School of Social and Political Science University of Edinburgh United Kingdom; ^2^ Head of the Department of Cognitive Science and Dean of the Faculty of Media and Knowledge Sciences University of Malta; ^3^ Researcher at the Department of Psychology University of Novi Sad Serbia; ^4^ Professor at the Department of Psychology University of Novi Sad Serbia

**Keywords:** Citizen Summits, community cohesion, culture, disaster preparedness, readiness, trust

## Abstract

This exploratory study investigates the relationships between the disaster preparedness of citizens and cultural factors in Romania and Malta. With regard to methodology, quantitative and qualitative data were collected during two Citizen Summits, which consisted of a real‐time survey and focus group discussions. The results point to two specific cultural factors that may bridge this ‘gap’ and be operationalised to enhance people's readiness for a disaster event. In Malta, the findings reveal how community cohesion is altered from a personal to a cultural value, which has the potential to encourage the transformation of preparedness intentions into actual preparedness behaviour. In Romania, meanwhile, the findings highlight the ambivalent aspects of trusting behaviour as a cultural norm on the one hand, and distrust in authorities based on experience and unmet expectations on the other hand. Social media use may reduce this tension between trust and distrust, and thus foster successful disaster risk‐related communication.

## Introduction

Appropriate disaster preparedness makes individuals and communities more resilient and reduces the negative consequences of an event (Becker et al., 2017). Models of preparedness (see, for example, Kirschenbaum, 2002) have indicated a number of factors that can influence the degree to which people prepare for a disaster, including: the extent to which they feel that they are in danger of experiencing a disaster (risk perception); their past experience of disasters; and the sociocultural context. Yet, it has proven difficult to find clear, unambiguous evidence of a connection between risk perception and disaster preparedness, as even people living in disaster‐prone areas are reported to have low levels of disaster preparedness (Kohn et al., 2012; Hoffmann and Muttarak, 2016). However, other research, such as that of Akompab et al. (2013), suggests that ‘culture’ may provide this missing link, as both risk perception and disaster preparedness are influenced by cultural factors such as local knowledge, rituals, values and norms, gender roles, collective memory, livelihoods, social cohesion, social exclusion, or trust in authorities.

The current study was carried out as part of the CARISMAND project,^2^ which sought to develop a ‘cultural toolkit'^3^ for disaster management stakeholders. The objective of this exploratory investigation, within the overall project framework, was to compare citizens’ attitudes, feelings, and perceptions in locations with different ‘disaster histories', focusing on the identification of specific cultural factors that may affect disaster preparedness and response in these locations. Here, ‘culture’ is taken to mean the shared beliefs, attitudes, values, and behaviours of a number of people,^4^ whereas ‘disaster preparedness’ is understood as measures taken by citizens to prepare for and to reduce the effects of an event, ranging from participation in formal training activities that are organised by experts to self‐organised, informal and personal measures, including the gathering of preparedness‐related information. The ‘disaster risk perception’ of citizens is seen to be composed of both cognitive (perceived risk/living in a disaster‐prone area) and emotional (worries/concerns about disasters in the area where the person lives) components.

Romania (high risk)^5^ and Malta (low risk)^6^ were selected as the research sites because they are countries with very dissimilar disaster risk profiles. The objective of gathering data from these two extremes on the risk continuum was to use the contrast between the two data sets to elucidate cultural factors that otherwise may remain unseen. In addition, Romania and Malta are located at the margins of Europe and, therefore, are more likely to be exposed to geopolitical changes. In such circumstances, people have been found to utilise cultural factors, especially values and traditions, as perceived stable elements to accommodate insecurity (Mitchell, 2002). The latter makes such locations particularly suitable for research on the associations between disaster risk perception, behaviour, and cultural factors. A mixed‐method approach was employed that combined quantitative measures of cognitive and emotional responses related to risk perceptions with qualitative methods to evaluate the dynamic character of culture vis‐à‐vis disaster preparedness.

## Disaster risk perception and preparedness

In the realm of disaster management, risk perceptions among affected populations have long been seen as determining the success of practitioners’ efforts to combat a disaster during all phases, from preparedness to response to recovery. Although the individual characteristics of people affected by disasters, or disaster hazards, model the outcome of each phase, it is important to recognise group similarities, given that these will help in improving the overall effectiveness of disaster management strategies. The literature supports the existence of demographic group differences: ethnic minorities (Olofsson and Rashid, 2011), women (Huang et al., 2013; Kaptan, Shiloh, and Önkal, 2013), and people with lower socioeconomic status (Fothergill and Peek, 2004) are shown to have higher levels of disaster risk perception than the rest of the population. Furthermore, there is evidence of group similarities beyond these demographic factors, suggesting a relationship between culture(s) and risk perception (see, for example, Lash, 2000; Lupton, 2006; Bora, 2007). A considerable number of studies explore these relationships, and methodological approaches range from quantitative and experimental research to qualitative case studies in cross‐cultural and social psychology, sociology, and social anthropology (see, for example, Bankoff, Frerks, and Hilhorst, 2004; Udayangani, 2010; Rohrmann, 2013; Wachinger et al., 2013; Krügeret al., 2015). Definitions of culture in these works rarely coincide, though, and they often still refer back to demographics, including ethnicity, geography, and socioeconomic factors, rather than cultural aspects, such as values and traditions, worldviews, power relations, and attitudes towards authorities. What is more, they frequently present contradictory results. For instance, living in a disaster‐prone area and past experience of disasters have been found to influence disaster risk perception, but these elements do not translate directly into a rise in future risk perception (Jones et al., 2013; He and Zhai, 2015), despite their potential to affect preparedness in a number of very specific ways^7^ (Becker et al., 2017). Nevertheless, the assumption that disaster risk perception directly alters disaster‐related behaviour, particularly those activities pertaining to disaster preparedness, has become increasingly contested (see, for example, Solberg, Rossetto, and Joffe, 2010).

Recent research suggests that cultural factors play a mediating role in disaster preparedness, as they are linked to both risk perception and behavioural adaptation (Akompab et al., 2013). However, there are cases that demonstrate how ambivalent these theoretical notions become when applied in practice. For instance, high levels of trust in authorities not only reduce risk perception, but also they can decrease engagement in preparedness activities (Grothmann and Reussig, 2006; Terpstra, 2011; Cornia, Dressel, and Pfeil, 2014). Trust in authorities incorporates a belief in the effectiveness of their efforts to prevent, prepare for, and respond to disasters, yet mistrust in their effectiveness does not necessarily lead to a rise in the preparedness activities of individuals or communities, owing possibly to fatalistic attitudes that may obstruct disaster preparedness (Paradise, 2005). Similarly, in a comparison of three highly seismic areas—Seattle, Washington, United States; Osaka, Japan; and Izmir, Turkey—Joffe et al. (2013) found that awareness of adaptive measures related to disaster risk reduction does not translate into adaptive behaviour if undermined by factors such as anxiety and distrust. At the same time, Islam and Walkerden (2014), based on the concept developed by Gittell and Videl (1998) and Putnam (2000), demonstrated that so‐called linking networks (vertical connections between citizens and organisations) do not necessarily produce adaptive behaviour. Consequently, one can argue that, although the cultural factor of ‘trust in authorities’ has good theoretical potential and explanatory power, it may be seen as a concept that is too complex when it comes to developing practical and successful disaster management guidelines. Instead, the cultural factor of ‘community cohesion’ may be better suited to the task, given previous findings. Community cohesion, sometimes in combination with the cultural value of family and extended family bonds in Mediterranean countries, was identified as having a positive bearing on a community's disaster resilience, promoting normative–supportive behaviour, fostering the sharing of information and resources, and contributing to swifter recovery from disasters (Lara et al., 2010; Patterson, Weil, and Patel, 2010; Joshi and Aoki, 2014). In this study, community cohesion comprises two dimensions, relating to (i) an individual's sense of belonging to a physical, or virtual, community,^8^ and (ii) the level of solidarity among the members of such a community.

However, the roles that community cohesion and trust in authorities may play in disaster preparedness are themselves subject to cultural differences. For example, in Romania, one of the most seismically‐active countries in Europe (UN ISDR, 2008), a study by Armaş, Crety and Ionescu (2017) revealed that those citizens who are less concerned about various natural or human‐made hazards, tend to trust an assortment of entities (such as the government, non‐governmental organisations (NGOs), or the fire department) less than those who do worry about disasters in the future. Yet, the same study research found no correlation between citizens’ trust in a particular entity and expected help from that institution. When aggregating these measurements for different bodies, though, Romanians who, on average, trusted institutions more, tended also to expect more support. Armaş, Crety, and Ionescu (2017, p. 13) concluded that at the ‘individual organisation level, there is no link between trust and expectancy, whereas at a more general level, one where the exact nature of the organisation matters less, and the person's psychology matters more, the link can indeed be found'. This finding suggests that trust in authorities may have different forms, or components, and should be investigated further in different contexts with different ‘histories’ of citizen–authority relations. In this paper, trust in authorities is understood as a cultural factor, which has multiple dimensions (trusting behaviour, trusting intentions, and willingness to trust) and relates to perceptions of competence and honesty.^9^

Disaster research has witnessed a rise in cross‐national comparisons in recent years (see, for example, Paton et al., 2010a, 2010b; Paton, Okada, and Sagala, 2013). Studies focus mostly on geographical areas that differ in terms of their level of hazard exposure (see, for example, Lindell and Prater, 2000, 2002; Lindell, Arlikatti, and Prater, 2009), types of hazard, or East–West concepts of collectivism versus individualism. Regarding the latter, research in cross‐cultural psychology and cultural neuroscience has produced contrasting results. The work is based on the notion of a ‘Western culture', encompassing characteristics such as independent social orientation and analytical information processing, rather than an ‘Eastern culture', representing interdependent social orientation and holistic information processing. Such assumed characteristics may explain certain differences (in perceived norm violation, for instance) in locations as disparate as the Japan and the US (Mu et al., 2015), but they do not easily elucidate behavioural differences within and between European countries.

Furthermore, research projects usually target areas with an increased hazard level, or where disasters have occurred recently. For example, Bucharest has been the site of several studies in the past decade on the relationships between socioeconomic factors, social vulnerability, and disaster risk perception (Armaş, 2007, 2008; Armaş and Avram, 2012; Armaş, Crety, and Ionescu, 2017). Yet, countries with a low prevalence of natural hazards, such as Malta, have rarely been investigated, with the exception of work on the perceived effects of climate change and health risk perception (Akerlof et al., 2010; DeBono, Vincenti, and Calleja, 2012). Research remains scant despite the fact that such ‘low‐risk’ countries can be struck by disasters triggered by human‐made risks as much as any of the ‘high‐risk’ countries. An interesting approach in this context was followed by De Pascale et al. (2015) who spotlighted the seismic risk perception of schoolchildren in Calabria, a high seismic‐hazard region in southern Italy, and the Mediterranean archipelago of Malta. In spite of their geographical proximity, these two locations are exposed to very different levels of (natural hazard) risk. In both cases, the results revealed a large gap among these children between risk awareness and knowledge of appropriate behaviour in the event of an earthquake, confirming once more that there are strong factors beyond disaster risk perception and disaster experience that affect disaster preparedness.

## Methods

Romania and Malta were chosen as research sites in order to compare high‐ and low‐risk locations with diametrically opposed ‘disaster histories’ and citizens with very different experiences of disasters. In addition, these two countries are located at the margins of Europe, and historically therefore, have been, and remain more likely to be, exposed to geopolitical changes and shifting influences, which can destabilise collective identities. Accordingly, anthropological research has found that people in such settings often use values and traditions as ‘stable elements’ in an environment that is perceived as potentially ‘insecure’ (Mitchell, 2002). This strong exposure of values and traditions makes them particularly suitable for an exploration of the cultural factors that affect behaviour.

### Citizen Summits

Empirical data for the current study were collected during one‐day public events, ‘Citizen Summits', organised in each of the two research locations. The term has its roots in public gatherings that are organised to ensure that ‘ordinary’ citizens, rather than experts or politicians, are given the opportunity to voice their opinions and to discuss issues of interest with the organisers. For example, the City Council in Washington, DC, US, held a Citizen Summit on 20 November 1999, at which residents were invited to review and provide input to plans for the budget request of 2001 and to comment on how the council delivers its services to communities. The initiative involved, inter alia, small focus group discussions led by facilitators and plenary sessions where participants used electronic keypads to provide immediate feedback (Callahan, 2006). This format has been followed in many similar events ever since, and Citizen Summits have been organised by myriad governmental institutions and NGOs on a variety of themes. Beyond being used to explore people's political priorities and to inform policymakers of which actions to implement, the concept of a Citizen Summit has also been employed more recently as a scientific research method, incorporating elements of quantitative and qualitative methodology in order to test theoretical models (see Degli Esposti and Santiago‐Gomez, 2015).

The CARISMAND Citizen Summits, during which the data for this exploratory study were procured, combined public information and public feedback‐gathering with quantitative and qualitative data collection.^10^ These two different methods were used to investigate the core aspects of this analysis: cultural factors, disaster preparedness, and risk perception. On the one hand, cultures are shaped by highly dynamic interconnected processes such as environmental and social changes, including media development, at the micro and macro level. At the same time, cultures play an important part in shaping individual and collective identity. Accordingly, they can have both destabilising and stabilising effects, as they comprise ‘cultural change’ and ‘cultural identity'. For a qualitative insight into these dynamics, focus group discussions were set up to identify shared narratives in which culture is situated. On the other hand, risk perception and attitudes towards disaster preparedness can be best explored by applying quantitative methods, such as in relation to the frequency and intensity of different types of perceived risk. Bringing these two methods together, the quantitative measures utilised in the current study were expected to provide a sound foundation for the further exploration of the qualitative linkages to the more ‘fluid’ cultural factors, which may shape disaster risk perception, disaster preparedness, and behaviour in disaster situations.

### Participants

Citizen Summit participants were recruited via local research agencies using a questionnaire. An industry‐standard ‘FreeFind’ approach was adopted, and participants were incentivised in line with regular practices for the research location concerned. The aim of the recruitment questionnaire was to achieve a balanced sample with an even gender and age distribution (see Table 1).

**Table 1:** Sample distribution by gender and age

**Citizen Summit location**	Gender				Age group (years)			
	Total	Female	Male	No answer	18–24	25–45	45+	No answer
**Romania**	**110**	54	51	5	22	42	44	2
**Malta**	**108**	50	53	5	19	44	41	4
**Total**	**218**	**104**	**104**	**10**	**41**	**86**	**85**	**6**

**Source**: authors.

Furthermore, the recruitment criteria included three key aspects of disaster experience and disaster risk perception (see Table 2), to ensure that all levels of experience of disasters were present in the sample.

**Table 2:** Recruitment criteria

	Answer=yes	
	Romania	Malta
**Experience of disasters**:	91.8%	50.0%
Have you, or a close friend or family member, ever experienced a disaster?		
**Feel that living in a disaster area**:	68.2%	38.0%
Do you feel you are living in an area that is specifically prone to disasters?		
**Know of vulnerable groups particularly exposed to disasters**:	78.2%	48.1%
Do you know of any other people in your area where you live who you think are particularly vulnerable or exposed to disaster?		

**Source**: authors.

The distribution of experience of disasters and risk perceptions in the sample in the two sites confirmed that the Romania sample can be considered as typical of a high‐risk location, whereas the Malta sample is more typical of a low‐risk location.^11^

### Procedure and materials

Each Citizen Summit was a day‐long event, and took place in a hotel in a central area to facilitate the travel arrangements of participants. The Romania Citizen Summit was held in the capital city of Bucharest, with participants recruited from the Bucharest metropolitan area. The Citizen Summit in Malta was held in St. Julian's, a centrally located town and traffic hub on the island, with participants recruited from all over Malta and neighbouring Gozo, the two largest islands within the Maltese archipelago.

Quantitative data were procured during two plenary sessions: one in the morning, and one at the end of the event. A total of 30 questions^12^ were put to the audience in four stages. The first stage consisted of an introductory presentation of the CARISMAND project, and a set of 11 initial questions, gathering demographic and other basic information on participants. In addition, their disaster preparedness intentions and their disaster risk perceptions were measured. Questions on risk perception were posed to the audience again in slightly different ways at later stages, each time after the provision of more disaster‐related information. The second stage featured videos and pictures of local disaster scenario exercises,^13^ followed by a second set of five questions that specifically requested participants’ views on such exercises, how well they felt informed about disaster preparedness, their intended behaviour in case of a high disaster risk, and, again, their risk perception. The third stage included a presentation on communication procedures in case of a disaster, followed by a third set of eight questions that targeted the perceived usefulness of social media in all disaster phases, and then a presentation on current social media use in disaster management.^14^ The fourth set of six questions, imparted in the afternoon, after the focus group discussions had been completed, concentrated, again, on the participants’ risk perception, but this time with a specific focus on different types of hazard. Immediate responses to all questions were captured via an audience response system.^15^ After each Citizen Summit, these responses were exported into a database and fully anonymised. All analyses were conducted using IBM SPSS for Windows, version 24.0, and significance tests were run for all results.

For the qualitative part of the study, participants were allocated to groups of between 9 and 11 people,^16^ with an even gender split. Questions posed in these focus group discussions built on the quantitative questions asked in the morning session, aiming to explore attitudes, feelings, and perceptions related to the same topics, but in a more open and intuitive way, and with a strong spotlight on relevant cultural factors. All of the focus groups were held in Romanian and Maltese respectively to avoid any language‐ or education‐related access restrictions and to allow participants to respond instinctively and to be able to discuss freely in their native tongue. All of the discussions were audio‐recorded and fully transcribed; the Romanian and Maltese transcripts were translated into English. To ensure the anonymity of the participants, all names and other personal identifiers were removed in the process. The coding of the translated transcripts adhered to a preliminary coding framework, which had been set up to permit an initial structuring of the collected data. This initial coding framework was based on 10 general themes defined in the focus group discussion guidelines. The results of this first coding allowed, in the next step, the development of a more refined matrix, with a total of 179 individual codes. After recoding the transcripts of all 20 focus group discussions based on this matrix, clusters were identified, which provided a better focus on specific processes and practices or constructions and interpretations. The qualitative results were compared with the quantitative results in a final step to paint a balanced picture, add depth, and increase the validity of findings.

## Results

### Quantitative findings: disaster preparedness, disaster risk perception, and disaster response

#### Disaster preparedness

The first part of this study focused in particular on the collection of quantitative data related to Romanian and Maltese participants’ disaster preparedness, disaster risk perception, and disaster response behaviour. Regarding disaster preparedness, participants at both Citizen Summits, especially those in Malta, expressed a strong lack of knowledge of the guidelines and procedures that their local disaster management authorities were following (Question (Q) 1.8; for the full questionnaire, see the Appendix). Almost three out of four (73 per cent) of Maltese respondents and slightly more than one‐half (55 per cent) of Romanian respondents indicated that they know ‘not a lot’ or ‘nothing at all'. Furthermore, participants also indicated that they felt even less informed about what to do themselves in case of a disaster, with 91 per cent of Maltese and 59 per cent of Romanian respondents feeling not informed or not informed at all (Q 2.3). The results of these two questions show a moderate correlation (Romania r_S_=0.45, p<0.001; Malta r_S_=0.41, p<0.001). There is a slightly stronger but still moderate relationship (Romania r_S_=0.49, p<0.001; Malta r_S_=0.51, p<0.001) between respondents feeling informed, or not informed, by the authorities on what to do, and feeling personally prepared for a disaster in their area (Q 1.10). Sixty per cent of Maltese and 35 per cent of Romanian participants indicated that they feel not prepared or not prepared at all, whereas only 9 per cent of Maltese and 22 per cent of Romanian participants felt prepared or well prepared.

**Figure 1:**
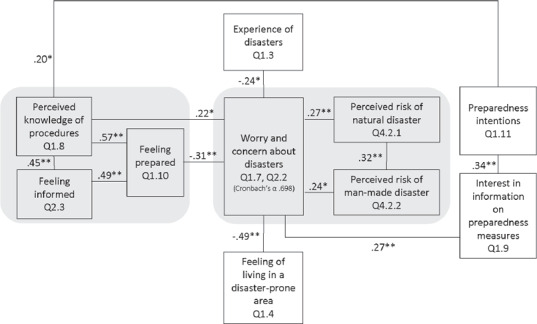
Citizen Summit, Romania: disaster preparedness and disaster risk perception **Notes**: The numbers shown denote the Spearman's Correlation Coefficient. ∗ Significance p<0.05; ∗∗ Significance p<0.001. **Source**: authors.

**Figure 2:**
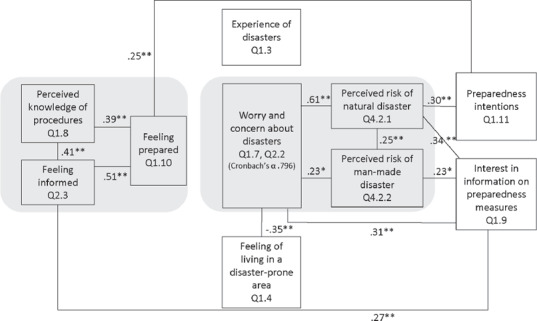
Citizen Summit, Malta: disaster preparedness and disaster risk perception **Notes**: The numbers shown denote the Spearman's Correlation Coefficient. ∗ Significance p<0.05; ∗∗ Significance p<0.001. **Source**: authors.

At the same time, though, participants expressed considerable interest in information on disaster preparedness. A large majority (85 per cent in Malta and 97 per cent in Romania) stated that they were quite or strongly interested in information on disaster preparedness (Q 1.9). In addition, 74 per cent of all participants signalled strong intentions to prepare for disasters (prepare quite a lot or a lot), with no significant difference between Romania and Malta (Q 1.11). Interestingly, the Romanian data show a significant correlation (see Figure 1) between participants’ interest in information on disaster preparedness and their intentions to prepare for disasters; there is no significant relation between these questions in the Maltese data (see Figure 2).

Neither the Romanian nor the Maltese data revealed any statistically significant differences between female and male responses with respect to all of the questions pertaining to disaster preparedness, or between different age groups.

#### Disaster risk perception

Disaster risk perception was measured^17^ at different points during the two Citizen Summits, in order to determine the potential influence of information and/or visual cues presented as videos and pictures of recent local disaster simulation exercises. Malta summit participants were shown a video rendering different scenes from a large‐scale simulation of a major earthquake (7.6 on the Richter scale, 120 kilometres southwest of Malta, 20 seconds in duration) on Gozo, the second largest of the inhabited islands in the Maltese archipelago. This simulation involved approximately 300 participants, including members of the Civil Protection Department, as well as police officers, soldiers, medical staff, and members of the general public.^18^ The videos shown during the Romania summit featured several simulation exercises, involving an accident at one of Bucharest's subway stations (2015), two aeroplane crashes (2015), and the explosion of a gas transport vehicle in the carpark of a large shopping mall, causing fire and a partial building collapse (2016).^19^ The results revealed a change in disaster risk perception after the simulation exercise were viewed. Having seen the videos, the number of participants who were worried/concerned about disasters in the area where they live increased significantly^20^ (see the responses to Q 1.7 and Q 2.2 in Table 3), particularly at the Malta summit. However, the rise in concern coincided: all Romanian participants (100 per cent) and a large majority of Maltese participants (87 per cent) found the simulations as shown to be important or very important (Q 2.1). This suggests that being aware of such exercises may augment the perceived risk of a disaster, but, at the same time, this appears to be seen as necessary and important.

**Table 3:** Disaster risk perception

	Romania		Malta	
	Mean	Standard deviation	Mean	Standard deviation
**Q 1.7: I am worried about disasters in the area where I live.**	3.92	0.89	2.93∗	1.12
**Q 2.2: When I think of disasters in my area, I feel concerned.**	4.23	0.77	3.45∗	1.02
Question posed after the showing of videos of disaster response exercises.				
**Q 4.2.1: I think there is a high risk of disasters triggered by natural hazards happening in my area in the next 3 years.**	3.32	0.82	2.62	0.88
**Q 4.2.2: I think there is a high risk of disasters triggered by man‐made hazards happening in my area in the next 3 years.**	3.43	1. 01	3.28	0.99
Questions Q 4.2.1 and Q 4.2.2 posed after the focus group discussions.				

**Notes**: answers measured on a five‐point Likert scale, ranging from 1=totally disagree to 5=totally agree. The results in the table marked with an asterisk (∗) signify that those between Romania and Malta are statistically significantly different (p<0.05). Other differences between Romania and Malta are not statistically significant.

**Source**: authors.

At the end of each Citizen Summit—that is, after the focus group discussions—participants were asked again for their risk perception, this time with a specific focus on the near‐to‐medium future (the next three years), and differentiating between the risks of disasters triggered by natural or human‐made hazards. The results show that only participants in Malta perceive a significant difference^21^ between the risk of disasters triggered by natural hazards and by human‐made hazards (see the responses to Q 4.2.1 and Q 4.2.2 in Table 3). Whereas 41 per cent of Maltese participants agree or totally agree that there is a high risk of disasters triggered by human‐made hazards, only 14 per cent agree or totally agree that there is a high risk of disasters triggered by natural hazards in their area in the next three years. More participants at the Romania Summit agreed than disagreed that there is a high risk of both in their area in the next three years. At the same time, a significantly lower number of Romanian participants^22^ perceived a high risk of disasters when asked at the end of the event (Q 4.2.1 and Q 4.2.2), as compared with the number of participants who were worried about disasters when asked at the beginning (Q 1.7). This suggest that more public information on and public discussion of disaster preparedness and response helps to reduce concern about disasters.

Figures 1 and 2 provide an overview of the various factors related to disaster preparedness and disaster risk perception. Differences in the relationships between them point towards different strategies in improving citizens’ preparedness in these different locations.

In Romania, a country with a rather high level of ‘objective’ and ‘subjective’ disaster risk, actual disaster experience (Q 1.3) appears to play a minor role in respondents’ worries and concerns about disasters. Romanian respondents who were interested in information on preparedness measures were moderately likely to intend to prepare for a disaster. Those who felt that they lived in a disaster‐prone area (Q 1.4) were also more likely to feel worried or concerned about a disaster. Interestingly, though, they were less likely to be interested in information on preparedness. The combination of these findings may point to a certain level of perceived helplessness, a phenomenon that was found in other research conducted with Romanian citizens on their ability to become active citizens.^23^ Such a phenomenon may best be addressed through communication with the general public that honestly presents the facts, and regular comprehensive information campaigns.

In Malta, a country with a rather low level of ‘objective’ disaster risk, participants indicated medium levels of subjective risk perception and feelings of worry/concern. These Maltese respondents’ preparedness intentions were directly related to specific perceptions of natural hazards, while their interest in information on preparedness measures was related to both disaster risk perception (natural and human‐made hazards) and worries/concerns, with these correlations being all rather weak. There was a strong link between perceived disaster risks (natural hazards) and worries/concerns. Disaster experience, however, was not connected to any of the other factors. This suggests that campaigns that aim to boost the level of Maltese citizens’ personal disaster preparedness may be more successful if they balance ‘objective’ facts with information that appeals to emotions.

There were no statistically significant correlations between the participants’ gender or age, and any of the questions presented in Figures 1 and 2, except for a weak‐to‐moderate relationship found in Malta between the participants’ age and their worry and concern about disasters (r_S_=0.30, p<0.001), and between their age and their interest in information on preparedness measures (r_S_=0.45, p<0.001).

#### Disaster response

The intended behaviour in disaster situations of Romanian and Maltese respondents was rather different (see Table 4). When asked what they would do *first* if there was a high risk of a disaster happening soon and they felt that it may cause serious harm, a majority of participants in the Malta Citizen Summit indicated that they would call their family and friends, whereas in the Romanian Citizen Summit, most said that they would call the emergency services. This result is consistent with the prominent role of family networks in Mediterranean societies (see, for example, Peristiany, 1976) and may be seen as a cultural factor that directly reflects behaviour in a time of disaster.

**Table 4:** First action in the case of a disaster

Imagine that a situation in which there is a high risk of a disaster happening soon, and you feel this disaster may cause serious harm to your family or friends. What is the first thing you would do?	Romania	Malta
Call the emergency services	69%	32%
Call family/friends	21%	54%
Go to your neighbours	1%	0%
Use social media to inform family/friends	1%	4%
Submit information via social media to authorities	1%	2%
Get more information via the internet	2%	3%
Get more information from social networks	0%	2%
Turn on the television/radio	1%	0%
Other/not sure/no answer	4%	4%

**Note**: there were no statistically significant differences between female and male responses, or between age groups.

**Source**: authors.

Although 93 per cent of the study participants indicated that they use social media, it appears that the vast majority in both countries do not turn to social media as their immediate response to an emergency (see Table 5). Only between two (Romania) and eight (Malta) per cent of participants stated that they would employ social media as their first response in an emergency, either to inform family/friends, submit information to authorities, or to gather more information. However, social media use was more likely in the event of an *ongoing* disaster. The largest proportion of Romanian participants would use social media to stay in contact with others (82 per cent likely or very likely), whereas the largest proportion of Maltese participants would use social media to inform themselves (80 per cent likely or very likely). The likelihood of submitting information to local authorities through social media was considerably lower. Nevertheless, 71 per cent of participants in Romania and 44 per cent in Malta described it as likely or very likely that they would use social media to submit information on disasters to the authorities, whereas 9 per cent in Romania and 35 per cent in Malta said it was unlikely or very unlikely.

**Table 5:** Social media use in disasters

Likelihood of using social media in case of an ongoing disaster for:	Romania		Malta	
	Mean	Standard deviation	Mean	Standard deviation
Informing oneself about the disaster	4.00	1.16	4.30∗	0.95
Submitting information on disaster risks/disasters to local authorities	3.93	0.94	3.19∗	1.32
Warning or informing other social media users	4.10	0.85	4.11	1.07
Warning or informing family and friends	4.14	1.11	3.86	1.20
Staying in contact with others	4.27	0.79	3.97∗	1.06
Providing help to others	4.15	1.00	3.73∗	1.15

**Notes**: answers measured on a five‐point Likert scale, ranging from 1=very unlikely to 5=very likely. The results in the table marked with an asterisk (∗) signify that those between Romania and Malta are statistically significantly different (p<0.05). Other differences between Romania and Malta are not statistically significant.

**Source**: authors.

These results suggest that the development of social media applications for disaster management should target multi‐functional solutions that allow different information flows, that is, authorities to citizens, citizens to other citizens, and citizens to authorities. They confirm, too, previous findings from outside Europe (Japan) where citizens perceived the availability of such multi‐level functionalities, and bridging these different levels of communication, as one of the most important characteristics of social media (Jung and Moro, 2014).

### Qualitative findings: cultures and cultural factors

In the first part of this study, questions to the general audience predominantly targeted attitudes towards preparedness and behavioural intentions. In contrast, the second part of the study concentrated more on actual behaviours related to disaster preparedness and response. Small focus group discussions were used to encourage participants to express their individual attitudes and experiences, as well as to observe specific group dynamics. These dynamics allowed for a particular focus in the data analysis on the potential influence of local cultures and cultural factors.^24^

Participants in both the Romanian and the Maltese groups tended initially to perceive disaster preparedness as the responsibility of governments, rather than reflecting on personal preparation measures. However, as the discussions progressed, the emphasis shifted noticeably from a perceived duty of public authorities and institutions to a more personal responsibility (see also Arlikatti, Lindell, and Prater, 2007). This shift also revealed a basic difference between the groups in the two summits. In Romania, participants described their disaster preparedness as predominantly information‐based and individualistic, being informed by or gathering information on procedures from authorities: ‘I took a disaster readiness class just before the holiday, and I also learned first aid, so it's also a matter of how open each person is'. Others included individual fitness as a means of preparation for disasters: ‘maintaining your own health and being healthy, not being ill'.

In Malta, rather than spotlighting individual actions, participants embedded their disaster preparedness more often in social relations and activities. They suggested improving preparedness through discussions with their families (such as about meeting points and means of communication in case of a disaster), planning to share resources among neighbours (such as sharing pumps in the event of flooded basements), and organising community meetings to discuss preparative measures. At the same time, Maltese participants portrayed their potential lack of preparedness as a specific ‘cultural trait’ related to living in pleasant surroundings: ‘Malta is an island […] and we are surrounded by so many beautiful things that we don't think of certain things [disasters]'; ‘as a nation […] we never think of negative things'; or ‘Malta is so small that the probability of an earthquake or these things hitting us is very remote. This is why we're a nation that doesn't worry'. Others pointed to other perceived cultural traits to explain a lack of preparedness: ‘[w]e do not prepare ourselves well, but Malta and the Maltese are very resourceful, and we get through disasters like in the wars^25^ in the past'.

In discussing their responses in disaster situations, Romanian and Maltese participants highlighted the need to keep calm, keep others calm, ensure the safety of family members, and offer voluntary help to the respective emergency services. However, a difference was found in participants’ self‐perception in terms of providing help. In the Maltese groups, the discussions revealed a general attitude that ‘everyone can help’ and that ‘everyone could use their skill set'. They suggested that ‘if someone is able to drive they can pick up a group of people and take them to hospital', or ‘a person who is like a builder', ‘perhaps an electrician', or ‘even simply leadership—in all that chaos you could be the person who does not panic and use that skill to help your family and those around you. That skill helps'.

In contrast, the participants in the Romanian groups described themselves as more ‘cautious', exercising ‘self‐control’ and trying not to obstruct or hamper the efforts of authorities, underlining that they would ‘offer services and obey directions'. At the same time, a number of them expressed their distrust in the authorities, relating it to a perceived lack of effectiveness in disaster response: ‘I notify the authorities but you can't be sure that they will come'. However, these participants simultaneously rationalised that such an attitude may be counter‐productive because ‘all expect help from the authorities but the authorities can't help if you don't trust them', referring to issues of perceived lack of effectiveness and dishonesty (corruption). This lack of trust may stem from personal experience and expectations not being met, which is at odds with a general trust in authorities derived from acceptance of hierarchical structures as a cultural norm. Accordingly, it would create ambivalent feelings about the relationships between citizens and authorities in disaster situations.

Most participants in the Romanian and Maltese Citizen Summits expressed a very strong willingness to help their fellow citizens with actions that blurred public and private spheres in such circumstances. Such willingness ranged from erecting tents in their private garden (Romania) to offering one's own kitchen to prepare food (Malta). However, a difference was revealed in the participants’ self‐perception. On the one hand, Romanian respondents described their approach as pragmatic and an individual attitude: ‘I went to donate blood', and ‘in the ‘77 earthquake I even dug graves'. On the other hand, Maltese participants explained such social solidarity as a cultural trait: ‘I think, here in Malta, thankfully our culture is to take care of each other', and ‘that's one of the virtues of the Maltese people: no matter how much they argue, they help'. This is a tradition that has persisted over time: ‘I think the Maltese community is divided in many things that, at the end of the day, become irrelevant. Be it politics, football, whatever, the village feast, but time and time again, even when financial help is needed, deep down the Maltese community is ready to help those around it'. This finding is particularly interesting not because community cohesion is seen as a Maltese cultural value, but rather because this value is being ‘operationalised’ by the participants for the specific purposes of disaster recovery and resilience.

In contrast, Bucharest summit participants referred predominantly to online communities, ‘whose members support and notify each other in case they notice that in the East, or in another country [a disaster has hit], […] not necessarily your next‐door neighbours, I mean through WhatsApp, Facebook, communicate with them and notify them because they might be the next ones hit'. Generally, and despite their explicit willingness to help other citizens in case of a disaster, the participants in the Citizen Summit in Romania appeared somewhat reluctant to ‘celebrate’ community cohesion in their own and immediate physical surroundings. Instead, they felt more at ease with imagining community cohesion in a virtual sense and space.

## Conclusion

Although more than two‐thirds of Romanian and one‐third of Maltese participants felt that they were living in a disaster‐prone area, both groups indicated low levels of knowledge of disaster guidelines or procedures, and they felt little informed or prepared. At the same time, they conveyed a very strong interest in information on preparedness measures, and strong intentions to prepare themselves for events in the future. These quantitative findings are consistent with the qualitative results, where the focus group discussion participants in both Citizen Summits expressed their desire for more information and training, and they made numerous suggestions to improve their knowledge and skills. However, the qualitative results also revealed cultural differences in preparedness‐related behaviour and preparedness intentions: Romanian participants focused mostly in the discussions on formal training and gathering information provided by the authorities, that is, measures that require direct guidance, whereas Maltese participants additionally outlined family discussions, community meetings, and neighbourhood help, that is, measures that are developed within their social networks.

In this context, the quantitative data gathered in the Malta Citizen Summit revealed a ‘disconnection’ between perceptions of disaster preparedness (feeling prepared for disasters and informed about the procedure to follow such events) and disaster risk perception (feeling worried or concerned about disasters), which confirms previous research (see, for example, Solberg, Rossetto, and Joffe, 2010). Furthermore, there are only weak correlations between perceptions of disaster preparedness and preparedness intentions and interest in information on preparedness. One possible explanation for this could be the generally low occurrence of large‐scale disasters in Malta's recent past, but it does not explain the same phenomenon among respondents in Bucharest. There, the lack of correlation between disaster risk perception and preparedness intentions may indicate the presence of barriers, which keep people with high perceived personal risk from acting (Weinstein and Nicolich, 1993). One of these barriers may be a perceived helplessness among the Romanian participants regarding their ability to become active citizens. These results address not only the known challenge for disaster managers that merely raising risk awareness does not translate into preparedness intentions, but also that preparedness intentions may not translate easily into preparedness activities. These are similar to the findings of Joffe et al. (2016) who demonstrated in their longitudinal intervention study that there is only a moderate correlation between perceived disaster preparedness and actual preparedness.

The strong sense of solidarity with others and the explicit hands‐on approach expressed by the Maltese participants may constitute a starting point in the identification of specific cultural factors that may prompt actual disaster preparedness behaviour. This point is further supported by collective memories that represent an important element of a group's collective identity. Yet, the interesting finding in this context is not the known linkage between community cohesion and disaster preparedness (see, for example, Lara et al., 2010), but that, in the focus group discussions, the Maltese participants reflected on both of these traits as a specific Maltese ‘culture to help'. At this point, it appears that social cohesion, understood as a shared value, is turned into a cultural norm, which elicits normative behaviour and may bridge the gap between preparedness intentions and preparedness actions (see also Appleby‐Arnold et al., 2018).

What is more, the qualitative and quantitative data collected in Malta highlight the prominent role of supportive behaviour in family networks in Mediterranean societies (see, for example, Peristiany 1976). The majority of Maltese participants stated that their first action in the event of a disaster would be to call their family and friends,^26^ whereas the majority of Romanian participants said that they would call the emergency services first. Both Romanian and Maltese participants indicated, though, that they would be very likely to make use of social media for various purposes in an ongoing disaster,^27^ involving different information flows: authorities to citizens (providing information); citizens to citizens (warning, helping, and staying in contact with others); and citizens to authorities. While interest in the latter functionality was not expressed as strongly as the other possibilities, more than two‐thirds of Romanian and almost one‐half of Maltese participants saw themselves as likely or very likely to submit information to the authorities during an ongoing disaster. This is consistent with the qualitative results that found a strong desire among the Romanian participants to ‘offer services and obey directions', despite their distrust in the authorities’ capability to respond effectively to disasters, and distrust owing to experiences of corruption. At the same time, some Romanian participants appeared to assume some of the responsibility themselves by acknowledging that the authorities can only act effectively when they have citizens’ trust. Implementation of social media applications for disaster communication may thus take advantage of the previous finding that strong bidirectional communications generally leads to a gradual reduction in tension between citizens and authorities (Busà et al., 2015), yet it also ‘bridges’ the gap between distrust in authorities as personal experience and trusting behaviour as a cultural norm.

Further research is needed, though, to assess how cultural factors, which have been identified as related to disaster preparedness intentions, can be sustainably transformed into agency. The findings of this exploratory study point in particular towards social cohesion and trust in authorities. These factors, which can be a cultural value and a cultural norm, should be tested in a longitudinal study, such as by measuring the efficacy of social media applications that make specific use of their different components in different experimental set‐ups and locations.

## Limitations

This research has some key limitations of which readers should take note. First, its cross‐sectional nature dictates that only correlation, not causation, could be studied. Accordingly, the lack of, or weak, associations between risk perception and preparedness intentions cannot be used to gauge the potential effects of perceptions on behaviour. Furthermore, the data in the two study locations were collected from non‐probability samples. Although they incorporated a spread of participants of all ages, an even gender split, and different levels of disaster experience and risk perception, the findings cannot be said to be representative of either the Romanian or Maltese population. The two locations were chosen owing to their different local disaster histories and types of local hazards, aiming to extract cultural factors in particular through the contrasting effect. However, the roles and functions of these cultural factors may differ in other locations, even if the locations have similar disaster histories and hazards.

## Appendix. Citizens Summit questionnaire

### Question set I: demographics, disaster preparedness, risk perception

**Table:** 

1.1	Gender (1=female, 2=male, 3=choose not to say)
1.2	Age (numeric)
1.3	Have you, or a close friend or family member, ever experienced a disaster?
	(1=yes, 2=no, 3=I'm not sure)
1.4	Do you feel you are living in an area that is specifically prone to disasters?
	(1=yes, 2=no, 3=I'm not sure)
1.5	Do you know of any other people in your area where you live who you think are particularly vulnerable or exposed to disasters?
	(1=yes, 2=no, 3=I'm not sure)
1.6	Do you work as a volunteer in a community or self‐help group?
	(1=yes, 2=no)
1.7	How much do you agree, or disagree, with the following statement: ‘I am worried about disasters in the area where I live'.
	(1=I totally disagree, 2=I disagree, 3=I neither disagree nor agree, 4=I agree, 5=I totally agree, 6=I'm not sure)
1.8	How much do you know about the guidelines and procedures your local disaster management authorities are following in case of a disaster?
	(1=nothing at all, 2=not a lot, 3=something, 4=quite a lot, 5=a lot, 6=I'm not sure)
1.9	To what extent are you interested in information about disaster preparedness?
	(1=not interested at all, 2=interested very little, 3=interested a little, 4=quite interested, 5=very interested, 6=I'm not sure)
1.10	How well do you personally feel prepared for a disaster in your area?
	(1=not prepared at all, 2=not prepared, 3=neither prepared nor unprepared, 4=prepared, 5=well prepared, 6=I'm not sure)
1.11	To what extent do you intend to prepare against disasters?
	(1=Prepare not at all, 2=Prepare very little, 3=Prepare a bit, 4=Prepare quite a lot, 5=Prepare a lot, 6=I'm not sure)

### Question set II: disaster communication

**Table:** 

2.1	What do you think about disaster simulation exercises like this^28^?
	(1=they are not important at all, 2=they are not important, 3=they are neither important nor unimportant, 4=they are important, 5=they are very important, 6=I'm not sure)
2.2	How much do you agree, or disagree, with the following statement: ‘When I think of disasters in my area, I feel concerned'.
	(1=I totally disagree, 2=I disagree, 3=I neither disagree nor agree, 4=I agree, 5=I totally agree, 6=I'm not sure)
2.3	How informed do you feel by the authorities of what you have to do in case of a disaster?
	(1=not informed at all, 2=not informed, 3=reasonably informed, 4=informed, 5=very informed, 6=I'm not sure)
2.4	Imagine that a situation in which there is a high risk of a disaster happening soon, and you feel this disaster may cause serious harm to your family or friends. What is the first thing you would do?
	(1=call the emergency services, 2=call family/friends, 3=go to my neighbours, 4=use social media to inform family/friends, 5=submit information via social media to local authorities/emergency services, 6=get more information via the internet, 7=get more information from social networks, 8=turn on the TV/radio, 9=other/I'm not sure)
2.5	What is the next thing you would do?
	(1=call the emergency services, 2=call family/friends, 3=go to my neighbours, 4=use social media to inform family/friends, 5=submit information via social media to local authorities/emergency services, 6=get more information via the internet, 7=get more information from social networks, 8=turn on the TV/radio, 9=other/I'm not sure)

### Question set III: use of social media in disaster situations

**Table:** 

3.1	Do you use social media?
	(1=yes, 2=no, 3=I'm not sure)
3.2	Do you use a mobile phone?
	(1=yes, 2=no)
3.3	In the case of an ongoing disaster, how likely are you to use social media to:
	3.3.1 inform yourself about the disaster
	3.3.2 submit information about disaster risks or disasters to local authorities/emergency services
	3.3.3 warn/inform other social media users
	3.3.4 warn/inform family and friends
	3.3.5 stay in contact with others during a disaster
	3.3.6 provide help to others during a disaster
	3.3.7 provide help to others?
	(1=very unlikely, 2=unlikely, 3=neither unlikely nor likely, 4=likely, 5=very likely, 6=I'm not sure)

### Question set IV: types of disaster, risk perception

**Table:** 

4.1	What do you think is the main cause for this^29^ disaster?
	(1=nature, 2=human activity, 3=both, 4=I'm not sure)
4.2	How much do you agree, or disagree, with the following statements:
	4.2.1 ‘I think that there is a high risk of a disaster triggered by natural hazards happening in my area in the next 3 years'.
	4.2.2 ‘I think that there is a high risk of a disaster triggered by man‐made hazards happening in my area in the next 3 years'.
	(1=I completely disagree, 2=I disagree, 3=I neither disagree nor agree, 4=I agree, 5=I completely agree, 6=I'm not sure)

**Source**: authors.

## Acknowledgements

The research reported in this paper was carried out as part of the CARISMAND project, which has received funding from the European Union's Horizon 2020 Research and Innovation Programme (2014–20), Grant Agreement Number 653748. The opinions expressed in this paper solely reflect the views of the authors; the EU is not responsible for any use that may be made of the information that it contains. The authors would like to thank Celia Callus at Nutcracker Research (United Kingdom) and Alexandra Tsvetkova at Libre Foundation (Bulgaria) for their contribution to the organisation of the two Citizen Summits, Jelena Radanović (University of Novi Sad, Serbia for her assistance with the review of literature on disaster risk perception for the CARISMAND project, and Iain Reid (University of Malta) for his assistance with analysing the qualitative data in Romania.

## Endnotes

2 The Culture And RISk management in Man‐made And Natural Disasters (CARISMAND) project received funding from the European Union under the Horizon 2020 Programme (2014–20), Grant Agreement Number 653748. For more information, see http://www.carismand.eu (last accessed on 13 October 2020).

3 This is an online knowledge base that includes a comprehensive set of guidelines and recommendations, mapping culture in the context of disasters. It can be used by disaster managers in their everyday practice, by policymakers as a basis for the development of disaster‐related policies and guidelines, and by interested citizens for awareness, information, and empowerment purposes. For more information, see http://www.carismand.eu (last accessed on 13 October 2020).

4 The CARISMAND project used the definition of ‘culture’ outlined by the International Federation of Red Cross and Red Crescent Societies: a set of ‘beliefs, attitudes, values and their associated behaviours, that are shared by a significant number of people in hazard‐affected places’ (IFRC, 2014, p. 14).

5 Romania is a country in southeast Europe that was part of the Soviet‐aligned Eastern Bloc. With a population of approximately 19.5 million, the post‐communist country has experienced a high rate of economic growth since 1989, although net average incomes are still among the lowest in the European Union. Bucharest, where the research was conducted, is the country's capital and largest city (with a population of roughly 1.9 million), and has among the highest rate of seismic risk of any city in the world (UN ISDR, 2008). The last devastating earthquake in Romania occurred on 4 March 1977. Measuring 7.2 on the Richter scale, it claimed 1,578 lives and injured an estimated 11,300 people, with 50 per cent of the casualties in Bucharest. Besides the prevalent high risk of natural hazards (earthquakes, flooding, and heat waves), citizens in the capital have also recently endured disasters triggered by human‐made hazards, such as the Colectiv nightclub fire on 30 October 2015, which resulted in 64 deaths and left another 146 people injured.

6 Malta is an island state in the central Mediterranean Sea; it is the smallest member of the European Union. With a population of approximately 500,000 spread over an area of 122 square miles, Malta is the tenth smallest and the fifth most densely‐populated sovereign country on the planet, with a highly industrialised, service‐based economy. The *World Risk Report 2016* (Jeschonnek et al., 2016) ranks it as the country with the second lowest level of general disaster risk in the world and the lowest in Europe.

7 Becker et al. (2017) describe seven different influences: (i) prompting thinking and talking; (ii) raising awareness and knowledge; (iii) helping individuals to understand the consequences of a disaster; (iv) developing beliefs; (v) developing preparedness; (vi) influencing emotions and feelings; and (vii) prompting community interaction on disaster issues.

8 The concept of ‘community’ has been widely criticised as being reductionist and implying unity, while hiding differences that may relate, inter alia, to gender or socioeconomic division (Mulligan et al., 2016). However, the authors believe that the use of the term in this study is justified, because it refers to the general involvement of citizens living in a specific location, but without assuming the homogeneity of such a local community.

9 The cultural factor of ‘trust in authorities’ was not predefined by the researchers, but emerged in the focus group discussions, where participants elaborated on their personal understandings, which coincide with a multi‐layered definition as outlined by PytlikZillig and Kimbrough (2016), among other authors.

10 The data on which this study is based were collected during the first two Citizen Summits. Overall, the CARISMAND project entailed six Citizen Summits, held in six different countries over the course of three years (year one: Malta and Romania; year two: Germany and Italy; and year three: Portugal and the Netherlands), and three ‘Stakeholder Assemblies', held in Romania (year one), Italy (year two), and Portugal (year three). The findings from each set of events shaped the content of the next, allowing therefore for a continuous cycle of knowledge exchange and the progression of ideas developed by disaster practitioners and citizens.

11 The high level of disaster experience and perceived disaster risk in Romania is consistent with the data published in the 2017 edition of the *World Risk Report* (as well as in the reports of previous years). Romania has an index indicating an elevated level of disaster risk among European countries, in combination with a comparatively high lack of coping capabilities and adaptive capacities. In contrast, Malta is ranked as second‐lowest in the world and lowest in Europe (Jeschonnek et al., 2017).

12 Questions related to sociodemographic information, disaster risk perception, and disaster preparedness were based, wherever possible, on previously established measures. These questions were posed during all six of the CARISMAND project's Citizen Summits. Questions pertaining to other topics (as well as qualitative questions), such as regarding social media use, had an exploratory character and were refined and/or amended at subsequent events.

13 Scenario exercises were organised by local disaster and emergency services for professional training purposes. These scenarios were related to either recent or likely incidents in the respective region, or due to their expected complexity. The exercises were not part of the CARISMAND project.

14 Presentations on specific topics, such as social media use in disasters, always took place after the respective set of questions to ensure that participants’ responses were not influenced by the respective information provided.

15 For more information, see http://www.clikapad.com (last accessed on 22 October 2020).

16 During each summit, two groups were composed of participants aged from 18–24 years, four groups were composed of participants aged from 24–44 years, and four groups were composed of participants aged more than 45 years. This division into age groups aimed to allow participants to enter into discussions with peers with similar life experiences.

17 To achieve adequate internal consistency but without using exactly the same wording, these questions are based on the five‐item measure developed by Kellens et al. (2011) with a Cronbach's alpha of 0.80 for the perception of flood risk, adapted to disasters in general.

18 The Civil Protection Department Malta, in cooperation with the Civil Protection Department Sicily and the Universities of Catania, Malta, and Palermo, conducted a complex disaster exercise in August–September 2015 as part of a co‐financed project with the European Union called the Integrated System for Transboundary Italo‐Maltese Civil Protection (SIMIT). Based on the video material provided by the Civil Protection Department Malta, a short film with several sequences was purpose‐cut for the Citizen Summit.

19 Videos and pictures were provided by the Romanian emergency rescue service, Serviciul Mobil de Urgenţǎ, Reanimare şi Descarcerare (SMURD).

20 Malta: t(86)=5.358; p=0.000; Romania: t(103)=3.585; p=0.001.

21 t(88)=5.649; p<0.001.

22 Q1.7/Q4.2.1: t(92)=5.148; p<0.001. Q1.7/Q4.2.2: t(94)=4.104; p<0.001.

23 Findings from the CONSENT (consumer sentiment regarding privacy on user generated content services in the digital economy) project (G.A. 244643), co‐financed by the European Union under the Seventh Framework Programme for Research and Technological Development, SSH‐2009‐3.2.1, ‘Changes in Consumption and Consumer Markets'.

24 Rather than providing participants with a pre‐set definition of the terms ‘culture’ and ‘cultural factors', the focus group moderators encouraged them to identify their own definitions and explanations, while specifically probing for factors beyond gender, age, socioeconomic status, or cultural stereotypes.

25 During the discussion session, several groups in Malta brought up the vast destruction in the areas around the Grand Harbour, which suffered intensive bombing during the Second World War of 1939–45 and has become part of Maltese collective memory.

26 A predominance of peer warnings is not unique to Mediterranean societies; they tend to be more frequent, too, in rapid‐onset disasters when authorities and news media outlets are unable to provide warnings before impact (Lindell, 2018; Lindell, Arlikatti, and Huang, 2019). However, the Maltese participants did not link their preference for peer warning to a perceived lack of effectiveness among disaster authorities.

27 For a comprehensive overview of the various uses of social media in all disaster phases, including their advantages and disadvantages, see, for example, Murthy and Gross; 2017; Jurgens and Helsloot, 2018; Reuter and Kaufhold, 2018; Appleby‐Arnold et al., 2019.

28 Relating to a presentation about a disaster simulation exercise that preceded this set of questions.

29 Participants were shown four pictures of different disaster situations: the earthquake in Ecuador in 2016; the flooding in Germany in 2016; the Fukushima Daiichi nuclear accident in Japan in 2011; and the heat wave in Europe in 2003.
